# Efficacy and safety of artemisinin-based combination therapy, and molecular markers for artemisinin and piperaquine resistance in Mainland Tanzania

**DOI:** 10.1186/s12936-018-2524-x

**Published:** 2018-10-17

**Authors:** Mwaka A. Kakolwa, Muhidin K. Mahende, Deus S. Ishengoma, Celine I. Mandara, Billy Ngasala, Erasmus Kamugisha, Johannes B. Kataraihya, Renata Mandike, Sigsbert Mkude, Frank Chacky, Ritha Njau, Zul Premji, Martha M. Lemnge, Marian Warsame, Didier Menard, Abdunoor M. Kabanywanyi

**Affiliations:** 1 Ifakara Health Institute, Dar es Salaam, Tanzania; 2 National Institute for Medical Research, Tanga Research Centre, Tanga, Tanzania; 3 Department of Parasitology, School of Public Health, Muhimbili University of Health and Allied Sciences, Dar es Salaam, Tanzania; 4 Catholic University of Health and Allied Sciences/Bugando Medical Centre, Mwanza, Tanzania; 5 National Malaria Control Programme (NMCP), Dar es Salaam, Tanzania; 6 World Health Organization Country Office, Dar es Salaam, Tanzania; 7 Aga Khan University Hospital, Nairobi, Kenya; 8 Gothenburg University, Gothenburg, Sweden; 9 Institut Pasteur, Paris, France

**Keywords:** Artemisinin-based combination therapy, Efficacy, Safety, Malaria, Molecular markers, Artemisinin, Piperaquine, Tanzania

## Abstract

**Background:**

Artemisinin-based combination therapy (ACT) is the first-line anti-malarial treatment of uncomplicated malaria in most malaria endemic countries, including Tanzania. Unfortunately, there have been reports of artemisinin resistance and ACT failure from South East Asia highlighting the need to monitor therapeutic efficacy of ACT in these countries as recommended by World Health Organization.

**Methods:**

Open-label single arm studies in mainland Tanzania were conducted in nine sentinel sites in 2011, 2012 and 2015 to assess the efficacy and safety of artemether/lumefantrine (AL) and artesunate/amodiaquine (ASAQ) using 28 days follow-up and dihydroartemisinin/piperaquine (DHAPQ) using 42 days follow-up. Mutations in the propeller domain of the *Plasmodium falciparum kelch 13* (*k13*) gene and amplification of the *P. falciparum plasmepsin 2* (*pm2*) gene, associated with artemisinin and piperaquine (PQ) resistance, were also investigated.

**Results:**

Of the 428 patients enrolled, 328 patients provided study endpoint. For AL, the PCR corrected per-protocol analysis showed adequate clinical and parasitological response (ACPR) of 90.3% (n = 28; 95% CI 74.2–98.0) in Kyela 2012, 95.7% (n = 22; 95% CI 78.1–99.0) in Chamwino, 100% in Muheza (n = 29; 95% CI 88.1–100), 100% in Nagaga (n = 39; 95% CI 91.0–100) and Kyela 2015 (n = 60; 95% CI 94.0–100). For ASAQ, PCR corrected ACPR of 98% (n = 49; 95% CI 89.4–99.9) and 100% (n = 25; 95% CI 86.3–100) were observed in 2011 in Ujiji and Kibaha, respectively. For DHAPQ, the ACPR was 100% (n = 71; 95% CI 94.9–100). Of the 235 samples with genetic interpretable results, only 7 (3%) had non-synonymous *k13* mutations. None of these are candidate or validated markers of artemisinin resistance and all patients carrying these alleles cleared the parasites on day 3. Of the DHAPQ group, 10% (3/29) of the samples with interpretable results had *pm2* multiple copies and none of them was associated with treatment failure.

**Conclusion:**

All the tested ACT in mainland Tanzania were highly efficacious and none of validated *k13* mutants associated with artemisinin resistance was observed. However, three isolates with multiple copy numbers of *pm2* gene associated with PQ resistance among the limited samples tested successfully calls for further investigation.

*Trial registration* Number ACTRN12615000159550. Registered 18th February 2015, https://www.anzctr.org.au/trial/MyTrial.aspx

## Background

Despite the global fall of malaria cases over the past decade, the disease still caused 6.8 million cases and 18,930 deaths in Tanzania in 2016 [1]. Malaria affects different age groups with children below 5 years carrying most of the burden [1]. *Plasmodium falciparum* is the predominant malaria species. Effective case management—early diagnosis and prompt treatment—is one of the pillars of malaria control and elimination. Following the World Health Organization (WHO) recommendation of artemisinin-based combination therapy (ACT) for the treatment of uncomplicated malaria, the Ministry of Health, Community Development, Gender, Elderly and Children of Tanzania introduced artemether/lumefantrine (AL) as first-line treatment for uncomplicated falciparum malaria in 2006 [2].

The emergence and spread of artemisinin resistance, as well as increased treatment failure (> 10%) with ACT in parts of South East Asia, underscores the need for malaria endemic countries to be vigilant and continuously evaluate the efficacy of the ACT medicines being used [3, 4]. WHO recommends monitoring the efficacy of the nationally used ACT every 2 years to inform national anti-malarial treatment policy to ensure effective treatment of malaria patients [5].

A review of nine studies previously conducted in different areas of Tanzania (in Muheza, Bagamoyo, Kibaha, Tabora, Mwanza, Kilombero, Zanzibar and Kyela) from 2002 to 2013 reported PCR corrected cure rate ranging from 88 to 94% for artesunate/amodiaquine (ASAQ) and 91 to 100% for AL. Only two studies reported cases of positive parasitaemia on day 3 which was below 2% [6]. Studies from East Africa and other parts of Africa also reported a high efficacy of ACT [7–15].

In vivo assessment of therapeutic efficacy of anti-malarial medicine is the gold standard for generating information to inform national malaria treatment policy. In addition detection of mutations or amplifications in *Plasmodium falciparum* genes linked to anti-malarial drug resistance confirms parasite resistance, supplementing the in vivo efficacy data. The discovery of point mutations in the *pfkelch13* (*k13*) gene and amplification of *pfplasmepsin2* gene provide opportunities to monitor artemisinin and piperaquine resistance, respectively [16, 17].

The current paper reports the findings of therapeutic efficacy studies conducted from 2011 to 2015, assessing the therapeutic efficacy and safety of AL, ASAQ and dihydroartemisinin/piperaquine (DHAPQ) in the mainland Tanzania.

## Methods

### Study design and areas

**Fig. 1:**
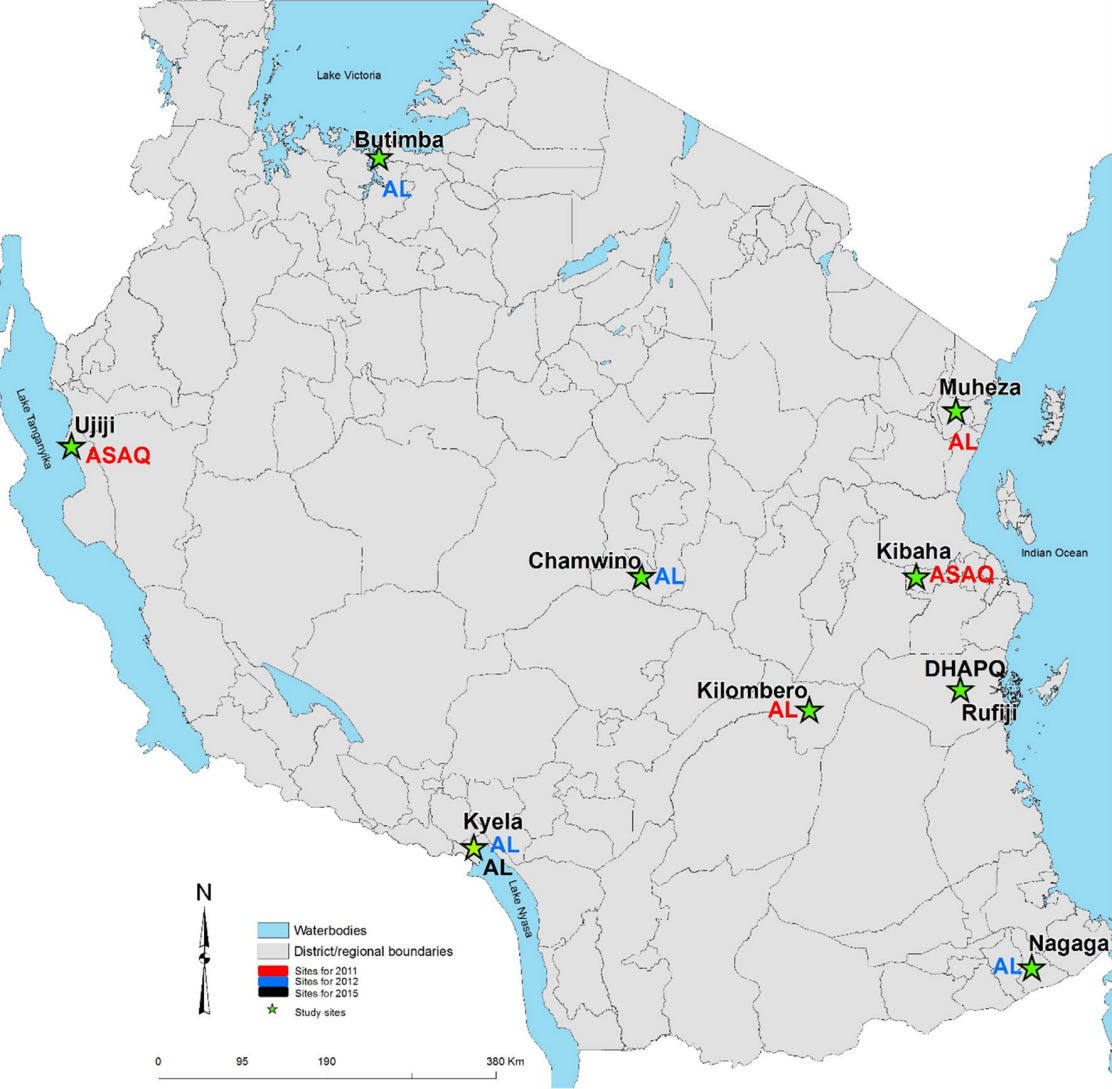
Map of Mainland Tanzania showing the location of the studies conducted in 2011 (red), in 2012 (blue) and in 2015 (black) by ACT treatment

Open-label, one-arm prospective studies (N = 8) were conducted to evaluate the clinical and parasitological responses to directly observed treatment of AL and ASAQ in 2011, AL in 2012, and AL and DHAPQ in 2015 using the WHO protocol for the surveillance of the efficacy of anti-malarial drugs [18]. The studies were conducted between March and September, covering the peak transmission season (June–August). They were carried out in the nine National Malaria Control Programme (NMCP) sentinel sites in eight regions; Chamwino in Dodoma, Butimba in Mwanza, Kibaha and Rufiji in Pwani, Kyela in Mbeya, Kilombero in Morogoro, Muheza in Tanga, Nagaga in Mtwara, and Ujiji in Kigoma (Fig. 1). Type of anti-malarial tested per site was agreed upon by all investigators ahead of protocol’s ethics approvals. In 2011 ASAQ was evaluated in Ujiji and Kibaha and AL in Kilombero and Muheza. Due to low number of patients enrolled in 2011, the Tanzania first-line anti-malarial (AL) was recommended to be assessed at all four sites (Butimba, Chamwino, Nagaga and Kyela) in 2012 while in 2015, AL and DHAPQ were evaluated only in Kyela and Rufiji, respectively.

### Participants and enrolment

Patients were enrolled if they had mono-infection of *P. falciparum* detected by microscopy, axillary temperature ≥ 37.5 °C or history of fever during the past 24 h, ability to swallow oral medication, ability and willingness to comply with the study protocol and visit schedule for the duration of the study and informed consent from the parent or guardian of the enrolled children. The age of the patients enrolled varied slightly in the different years: 6–59 months in 2011; 6 months–10 years in 2012 and 6 months and above in 2015. Similarly, the threshold of parasitaemia at enrolment varied from 1000 to 200,000 asexual/µl asexual forms in 2011 and in 2012 to 250–200,000 asexual/µl in 2015. WHO protocol allows to adjust age and enrolment parasitaemia of study patients according to the transmission levels [18]. ACT is contraindicated in the first trimester of pregnancy and, therefore, the WHO protocol requires that all females who reached menarche to be tested for pregnancy. For the study in 2015 female minors aged 12–17 years were excluded from the study as confidentiality and privacy required for pregnancy testing for this age group could not be ensured. Any of the following features was regarded as an exclusion criteria: presence of general danger signs or signs of severe falciparum malaria according to the definitions of WHO, severe malnutrition, febrile conditions due to diseases other than malaria or other known underlying chronic or severe diseases, regular medication, which may interfere with anti-malarial treatment, history of hypersensitivity reactions or contraindications to any of the medicine(s) being tested or used as alternative treatment(s).

At the enrollment, home address, demographic information and full medical history were taken from each patient. Body weight was measured and axillary temperature was taken. Physical examination was done to rule out danger signs and other non-malaria illnesses as per exclusion criteria.

### Treatments and follow-up

All eligible patients were treated with oral AL, ASAQ or DHAPQ using the WHO recommended therapeutic dose regimens [19]. All treatment doses were given under direct supervision of the study clinician or study nurse in health facilities and participants were observed for 30 min for adverse reactions or vomiting. For AL arm, participants residing far from the health facilities were retained at the facility for the night doses and allowed to go home in the morning. The daily dose was re-administered in case of vomiting within 30 min after drug intake, one child was withdrawn from the study and given the rescue medication after vomiting the replacement dose. Children with fever ≥ 38.5 °C were treated with paracetamol and tepid sponging was also used for those below 5 years. Patients were assessed clinically and parasitology according to the WHO protocol [18] and were followed up for 28 days for treatment with AL and ASAQ, and 42 days for DHAPQ.

### Laboratory assessment

Thick and thin smears were taken on days 0 (before treatment) and during subsequent prescribed follow-up on day 1 (if presented with danger signs or severe symptoms), 2, 3, 7, 14, 21, 28 days in 28 days for AL and ASAQ whereas for DHAPQ up to day 42 days. Microscopy examination of thick and thin blood films using Giemsa stain was undertaken to determine parasite species and density according to the WHO protocol [18]. Parasite density (per µl) was calculated assuming a white blood cell count of 8000/µl. All slides were read independently by two qualified microscopists and the average of the two counts was taken as the parasite density. Parasite counts with discordant results (differences in species, presence of parasites or parasite density > 50%) were re-read by a third independent microscopist, and parasite densities were calculated by averaging the two most close counts.

Two to three drops of blood were collected on filter paper (Whatman no. 3 mm, GE Healthcare UK Limited, UK) on day 0 (before treatment) and during recurrence of parasitaemia on day 7 onwards. Each dried blood spots (DBS) was punched with a sterile puncher and placed in a 96 well plate in numerical order. Samples were lysed overnight in a saponin solution, and then, DNAs were extracted with the Instagen Matrix resin as previously described [20]. All DNA samples from patients undergoing symptoms reoccurrence were analysed for genotyping of the highly polymorphic regions *msp1*, *msp2* and *glurp* loci, as recommended by WHO [21]. DNA samples were also analysed for the presence of mutations in the propeller domain in the *k13* gene associated to artemisinin resistance [16]: a portion of the *k13* gene was amplified using a nested PCR assay, amplicons (codons 443–666, i.e. 720 bp) were sent to Macrogen Korea for sequencing, and DNA sequences were analysed to identify specific single-nucleotide polymorphism (SNP) related to artemisinin resistance. Samples from the DHAPQ treated patients were also analysed for amplification of *plasmepsin2* gene recently associated to piperaquine resistances [17].

### Treatment outcome

Treatment outcomes were determined according to the classification of WHO [18] as early treatment failure (ETF), late clinical failure (LCF), late parasitological failure (LPF) and adequate clinical and parasitological response (ACPR). The proportion of patients who were still positive on day 3 was also recorded. Following PCR correction, treatment failure was classified as recrudescence if recurrent parasitaemia was the same parasite strain as of day 0 or new infection if they were different parasite strain. Adverse events including serious adverse events were assessed through physical examination and history taking.

### Sample size and statistical analysis

An expected treatment failure rate of 5% was assumed for all the three drugs evaluated and with a 95% confidence level and a precision of 5%, a sample size of 73 was targeted per site or per drug for 2011, 2012 and 2015 studies. An additional 20% was added to ensure that the sample size would be achieved after the exclusion of patients due to loss to follow-up or withdrawal. A total of 88 patients per drug per site were therefore targeted. Data were double entered and analysed using the WHO-designed Microsoft Excel^®^ program (Microsoft Corporation, Washington). Chi-square test or the Fisher’s exact test (where cell counts were ≤ 5) were used for comparing differences in categorical data. Arithmetic and geometric (only for parasite density) means were considered.

Proportions of treatment failures (ETF, LCF and LPF) and cure rates before and after PCR correction were calculated as per-protocol analysis and Kaplan–Meier analysis. Re-infections and non-interpretable PCR results were excluded from the per-protocol analysis, but retained in the Kaplan–Meier analysis.

### Ethics approvals and registration

These studies were approved by the Ifakara Health Institute (IHI) ethics review committee (ERC), the National Medical Research Coordinating Committee (MRCC) of the National Institute for Medical Research (NIMR) and by the WHO research ethics review committee for studies conducted in 2015.

All English consent forms were translated into Swahili and back translated by a native speaker. Before enrollment, parents or guardians of children below 18 years were asked for a written informed consent. In addition, an assent was sought for all children between 12 and 17 years. For illiterate parents or guardians, a literate witness who was not part of the study team was chosen to sign on his or her behalf. All the information obtained was kept confidential and shared with study team only when it was necessary. Identification numbers were used instead of patients’ names in the case report forms and computer based data entry.

## Results

**Fig. 2:**
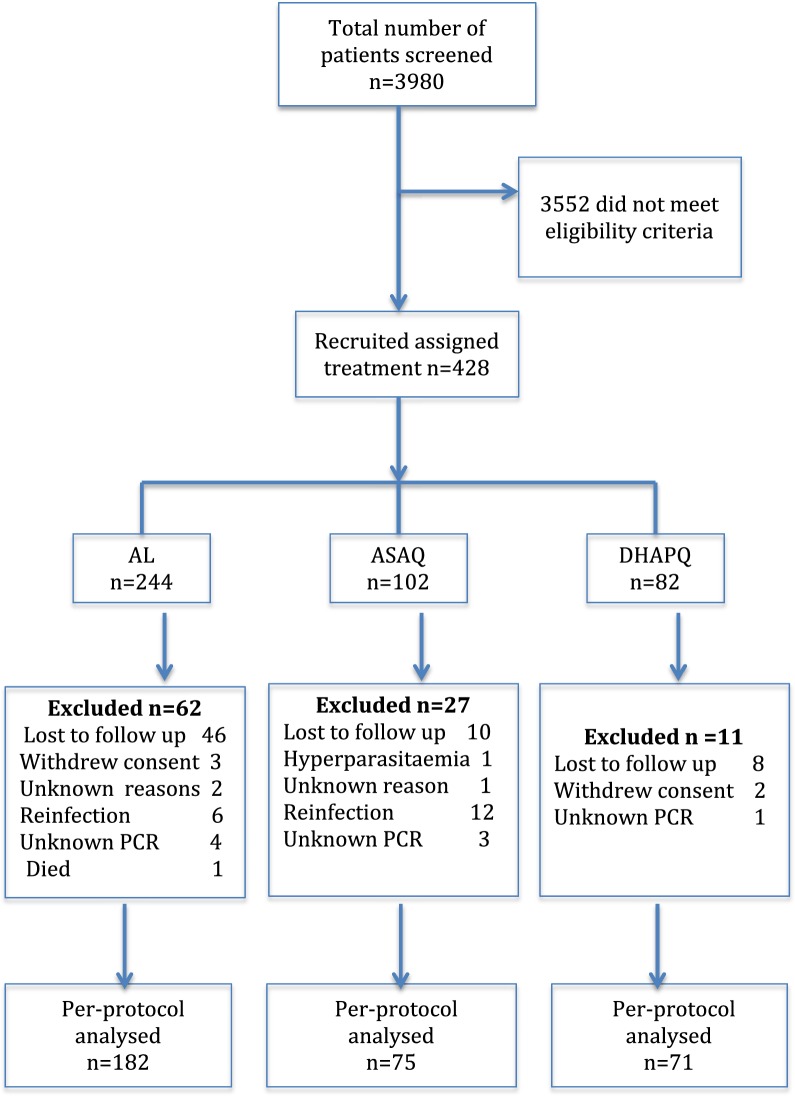
Flow chart: *ASAQ* artesunate + amodiaquine, *AL* artemether + lumefantrine, *DHAPQ* dihydroartemisinin + piperaquine

The baseline characteristics of all study patients are summarized in Table 1. A total of 3980 patients were screened and only 428 (11%) met criteria for enrollment (Fig. 2). There was no significant difference in sex and body temperature while there was variation of geometric mean of parasite density across the sites. In addition, the age profile of the study patients varied between the years ranging from 5 years in 2011, up to 10 years in 2012 and all ages in 2015 based on the inclusion criteria for each year.

**Table 1:** Baseline characteristics of the study cases at enrollment from the different sites

Year	Drug/site	Enrolled numbers	Age (years) Median (range)	Sex (male) n (%)	Temperature (°C) Mean (SD)	Parasitaemia (μl) GM (range)
2011	ASAQ					
	Ujiji	73	2.7 (0.2–5)	37 (50.7)	38.4 (1.3)	48,183 (1600–198,194)
	Kibaha	29	3.0 (1–5)	17 (58.6)	38.2 (1.1)	6162 (1034–80,000)
	AL					
	Muheza	32	2.0 (1–5)	18 (56.3)	37.9 (1.2)	24,400 (1680–160,457)
2012	AL					
	Chamwino	26	5.0 (1–8)	13 (50.0)	38.1 (1)	8146 (1120–36,800)
	Kyela	44	3.0 (1–10)	20 (45.5)	38.1 (1.2)	32,741 (1680–180,800)
	Nagaga	62	3.0 (1–10)	30 (48.4)	38.3 (0.9)	22,368 (1040–149,382)
2015	AL					
	Kyela	80	6.5 (0.6–60)	40 (50.0)	38.1 (1.3)	13,303 (640–84,480)
	DHA-PQ					
	Rufiji	82	10 (0.8–87)	44 (51.2)	37.6 (0.9)	12,007 (960–199,120)

*GM* geometric mean, *ASAQ* artesunate/amodiaquine, *AL* artemether/lumefantrine, *DHA-PQ* dihydroartemisinin/piperaquine

Tables 2 and 3 present the treatment outcomes by year, site and drug. A total of 64 patients were lost during the follow-up, 18 re-infections and 5 were excluded after enrolment due to consent withdrawal. PCR non-corrected ACPR varied from 75 to 100% and 78 to 100% for ASAQ and AL, respectively (Table 2), while 99% of the patients treated with DHAPQ achieved ACPR. No ETF was observed. All study patients cleared their parasites by day 3 except two cases (6.3%) treated with AL in Muheza. PCR corrected ACPR was 98% and above for ASAQ, 90% and above for AL and 100% for DHAPQ (Table 3).

**Table 2:** Treatment responses in patients treated with artesunate/amodiaquine, artemether/lumefantrine and dihydroartemisinin/piperaquine in Tanzania: PCR uncorrected

Year	Drug/site	N	PD3	Excluded/lost	LCF	LPF	ACPR	Kaplan–Meier
			n (%)	n (%)	% (95% CI)	% (95% CI)	% (95% CI)	% (95% CI)
2011	ASAQ							
	Ujiji	73	0	8 (11.0)	13.8 (6.5–24.7)	10.8 (4.4–20.9)	75.4 (63.1–85.2)	76.1 (64.0–84.6)
	Kibaha	29	0	4 (13.8)	0 (0.0–13.7)	0 (0.0–13.7)	100 (86.3–100)	100
	AL							
	Muheza	32	2 (6.3)	2 (6.3)	0 (0.0–11.6)	3.3 (0.1–17.2)	96.7 (82.8–99.9)	96.9 (79.8–99.6)
2012	AL							
	Chamwino	26	0	3 (11.5)	0 (0.0–14.8)	4.3 (0.1–21.9)	95.7 (78.1–99.9)	95.7 (72.9–99.4)
	Kyela	44	0	8 (18.2)	5.6 (0.7–18.7)	16.7 (6.4–32.8)	77.8 (60.8–89.9)	78.9 (62.3–88.9)
	Nagaga	62	0	23 (37.1)	0 (0.0–9.0)	0 (0.0–9.0)	100 (91.0–100)	100
2015	AL							
	Kyela	80	0	16 (20.0)	4.7 (1.0–13.1)	1.6 (0.0–8.4)	93.8 (84.8–98.3)	94.1 (85.0–97.8)
	DHA-PQ							
	Rufiji	82	0	10 (12.2)	1.4 (0.0–7.5)	0 (0.0–5.0)	98.6 (92.5–100)	98.6 (90.8–99.8)

*PD3* positive on day 3, *LCF* late clinical failure, *LPF* late parasitological failure, *ACPR* adequate clinical and parasitological response, *ASAQ* artesunate + amodiaquine, *AL* artemether + lumefantrine, *DHAPQ* dihydroartemisinin + piperaquine

**Table 3:** Treatment responses in patients treated with artesunate/amodiaquine, artemether/lumefantrine and dihydroartemisinin/piperaquine in Tanzania: PCR corrected

Year	Drug/site	N	Excluded/lost	Re-infection	Uknown	LCF	LPF	ACPR	Kaplan–Meier
			n (%)	n (%)	n (%)	% (95% CI)	% (95% CI)	% (95% CI)	% (95% CI)
2011	ASAQ								
	Ujiji	73	8 (11.0)	12 (16.4)	3 (4.1)	0 (0.0–7.1)	2 (0.1–10.6)	98 (89.4–99.9)	98.3 (88.8–99.8)
	Kibaha	29	4 (13.8)	NA	NA	0 (0.0–13.7)	0 (0.0–13.7)	100 (86.3–100)	100
	AL								
	Muheza	32	2 (6.3)	0	1 (3.1)	0 (0.0–11.9)	0 (0.0–11.9)	100 (88.1–100)	100
2012	AL								
	Chamwino	26	3 (11.5)	0	0	0 (0.0–14.8)	4.3 (0.1–21.9)	95.7 (78.1–99.0)	95.7 (72.9–99.4)
	Kyela	44	8 (18.2)	3 (6.8)	2 (4.5)	6.5 (0.8–21.4)	3.2 (0.1–16.7)	90.3 (74.2–98.0)	91.5 (75.9–97.2)
	Nagaga	62	23 (37.1)	NA	NA	0 (0.0–9.0)	0 (0.0–9.0)	100 (91.0–100)	100
2015	AL								
	Kyela	80	16 (20.0)	3 (3.8)	1 (1.3)	0 (0.0–6.0)	0 (0.0–6.0)	100 (94.0–100)	100
	DHA-PQ								
	Rufiji	82	10 (12.2)	0	1 (1.2)	0 (0.0–5.1)	0 (0.0–5.1)	100 (94.9–100)	100

*Unknown* unknown based on PCR analysis, *LCF* late clinical failure, *LPF* late parasitological failure, *ACPR* adequate clinical and parasitological response, *ASAQ* artesunate/amodiaquine, *AL* artemether/lumefantrine, *DHAPQ* dihydroartemisinin/piperaquine

### K13 mutations

Among 428 recruited patients, 324 day 0 samples were available and analysed for *k13* mutations. Out of these, 235 samples gave interpretable sequence and the remaining 89 samples were either PCR negative (n = 51) or gave non-interpretable data (n = 38). Out of the samples with interpretable data, 93.6% (n = 220) had wild-type *k13* alleles. The remaining carried, non-synonymous *k13* mutations (3.0%, n = 7) and synonymous *k13* mutations (3.4%, n = 8). The non-synonymous *k13* mutants observed were L463S, G496S, M476I, V510M, E556K, M562T and E602D. None of them was associated with day 3 positive parasitaemia and they are not among the known *k13* mutants associated with artemisinin resistance. Of note, the M476I mutant, previously associated with in vitro artemisinin resistance in a similar Tanzania strain (F32-ART) was observed once in 2012 in a patient treated with AL from Kyela site. Though the patient was lost follow up on day 21, all the parasites were cleared up by day 3.

### *Plasmepsin2* gene amplification

All the DHAPQ (82/82) D0 samples were available for *pm2* gene amplification analysis. Of these, 29 (35.4%) samples gave interpretable data of which 3 (10.3%) had two copies *pm2* and all had ACPR. The sequences of the remaining 53 (64.6%) samples gave non-interpretable results due to too low amount of DNA or poor quality of DNA extracts.

### Adverse events

Few non-serious adverse events were reported across the sites, the most common were cough 23/428 (5.4%), vomiting 10/428 (2.3%), fever 4/428 (0.9%) and diarrhoea 4/428 (0.9%). One participant from Chamwino vomited AL twice, he was withdrawn from the study and was given quinine injection as a rescue treatment. A death of a female child aged 6 years from Nagaga site was reported. The information regarding the possible cause of death was not known, the child attended the first two visits only.

## Discussion

The findings showed PCR-corrected cure rate of 90% and above for AL, 98% and above for ASAQ and 100% for DHAPQ treatments in the study areas between 2011 and 2015. Treatment with AL, the first-line drug for treatment of uncomplicated falciparum malaria in Mainland Tanzania, achieved ACPR above 95% except in 2012 in Kyela where ACPR was 90% the borderline below which WHO recommends ACT should be replaced [19]. However, the result from the same site 3 years later showed cure rate of 100% for the same ACT medicine. Since all patients, including under-five children (n = 35), achieved ACPR in 2015, the higher treatment response in 2015 could not be attributed to the inclusion of older children (n = 34) and adults (n = 11), population groups with potentially higher immunity to enhance treatment outcome.

AL is the first-line treatment of choice in most of the malaria endemic African countries and has remained highly effective after more than a decade of its use [7–15]. In Tanzania AL is still highly efficacious despite the fact that mutations in *mdr1* codons have been detected and showed increased trend [22, 23].

Anti-malarial ASAQ is not included in the national first-line treatment policy of the mainland Tanzania, although it is widely available in the private sector and is the first-line treatment in Zanzibar [1], which is part of the United Republic of Tanzania. This study gave reassurance that this ACT medicine is highly efficacious for the treatment of uncomplicated falciparum malaria. Similar high cure rate has been reported from other African countries [7, 9, 12, 14, 15, 24, 25].

The high cure rate of DHAPQ in this study supports earlier findings from and across Africa [11, 15, 24, 26–28]. This ACT medicine is being increasingly recommended in malaria endemic countries as second-line treatment for falciparum malaria and also used for mass drug administration in special situations. However, the emergence of piperaquine resistance as well as high treatment failure with DHAPQ from several countries in South East Asia calls for continuous monitoring in countries using this ACT.

Suspected artemisinin resistance is defined as ≥ 10% of patients with persistent parasitaemia by microscopy at 72 h (day 3) after treatment with ACT or artesunate monotherapy or ≥ 10% of patients with a half-life of the parasite clearance slope ≥ 5 h after treatment with ACT or artesunate monotherapy or ≥ 5% of patients carrying *k13* resistance-confirmed mutations [29]. Artemisinin resistance is confirmed if ≥ 5% of patients carrying *k13* resistance-confirmed mutations and that patients have either persistent parasitaemia by microscopy on day 3 after treatment with ACT or artesunate monotherapy, or a half-life of the parasite clearance slope ≥ 5 h [29]. In the current study, the lack of *k13* validated mutants [29] supported by the clearance of parasitaemia by day 0 in all patients might suggest absence of suspected artemisinin resistance in *P. falciparum* in the study areas. This finding is in line with previous report from Africa [30]. So far, several non-synonymous *k13* alleles have been reported in different parts of Africa, none of which has been linked to alleles from South-East Asia neither associated with day 3 parasitaemia or delayed parasite clearance [31–34]. The M476I mutant, found in one patient from Kyela in 2012, was not observed in studies conducted the same site in 2015.

The recent evidence provided by Witkowski and colleagues [17] and Amato et al. has shown a high association between the *pm2* multiple copies and piperaquine resistance in Cambodia [35]. DHAPQ treatment failure, associated with increased *pm2* multiple copies, was reported from several countries in South East Asia [17]. The findings of 10% cases with *pm2* copies (n = 2) among the limited samples (n = 30) successfully analysed, indicate the possibility of presence of parasites resistant to piperaquine in Tanzania and calls for further studies with adequate sample size. Recently low prevalence of *pm2* multiple copies has been reported from Mozambique [36].

In this study, few mild adverse events (a cough, fever, diarrhoea, vomiting and others) were reported and almost all resolved soon after completion of the treatment. Similar tolerable adverse events have been associated with ACT, the most common being cough, fever, vomiting followed by gastrointestinal disturbances [15, 37].

## Limitation of the study

Although this study attained sample size below 50, minimum sample to give interpretable results, in Kibaha, Muheza, Chamwino, Kyela in 2011/2012, the results from the remaining sites show that ASAQ, AL and DHAPQ are highly effective in the treatment of uncomplicated falciparum malaria Mainland Tanzania.

Several study sites failed to enroll the target sample size of 73 cases per site per drug and four of the sites enrolled the minimum 50 cases, a sample which provides interpretable outcome. The low number of enrollments in 2011 and 2012 may be explained by the variation in seasonality and temporal downturn of malaria cases in most parts of Tanzania Mainland although resurgence was recorded afterwards from 2013 onwards [38, 39].

A large number of D0 filter paper blood samples were not available, either lost or used for parasite genotyping to differentiate reinfection from recrudescence. In addition a substantial number of the available blood spots gave either negative PCR results or non-interpretable sequences, likely due to the low amount of parasite DNA or the degradation of DNA during storage or both as samples collected in 2011–2015 were analysed end of 2017.

## Conclusion

The findings documented that all the tested ACT medicines were highly efficacious, including AL despite its introduction for more than a decade, and lack of *k13* validated mutants. However, the presence of parasites with *pm2* gene amplification among the limited samples tested successfully calls for further investigations. Lastly, the population selected in this study represents the high to low transmission areas in mainland Tanzania and the most vulnerable age group for uncomplicated malaria cases.

## Abbreviations

ACT: artemisinin-based combination therapy
ACPR: adequate clinical and parasitological response
AL: artemether/lumefantrine
ASAQ: artesunate/amodiaquine
DHAPQ: dihydroartemisinin/piperaquine
DNA: deoxyribonucleic acid
ETF: early treatment failure
IHI: Ifakara Health Institute
LCF: late clinical failure
LPF: late parasitological failure
*mdr1*: *Plasmodium falciparum multi drug resistance1*
MRCC: Medical Research Coordinating Committee
NMCP: National Malaria Control Programme
PCR: polymerase chain reaction
SNP: single-nucleotide polymorphism
WHO: World Health Organization
